# An Atypical Presentation of Crohn's Disease: A Case Report

**DOI:** 10.7759/cureus.29431

**Published:** 2022-09-21

**Authors:** Asfand Yar Cheema, Mishaal Munir, Kaneez Zainab, Oboseh J Ogedegbe

**Affiliations:** 1 Medicine, Services Hospital Lahore, Lahore, PAK; 2 Internal Medicine, Lahore Medical & Dental College, Lahore, PAK; 3 Medicine, Ghurki Trust & Teaching Hospital, Lahore, PAK; 4 Internal Medicine, Mayo Hospital, Lahore, PAK; 5 Internal Medicine, Lifeway Medical Center, Abuja, NGA

**Keywords:** intestinal tuberculosis, crohn’s disease (cd), ulcerative colitis (uc), intestinal lymphoma, inflammatory bowel disease

## Abstract

Crohn’s disease (CD) is an inflammatory bowel disease affecting any portion of the gastrointestinal tract, usually the terminal ileum and the colon, with clinical manifestations such as diarrhea, fever, and weight loss. Clinical presentation of CD may include complications such as enterovesical fistulas, abscesses, strictures, and perianal disease. CD also classically presents with “skipping lesions,” unlike ulcerative colitis (UC), which presents with continuous lesions. It can manifest with a wide range of extra-intestinal symptoms such as pyoderma gangrenosum, aphthous stomatitis, episcleritis, uveitis, and arthritic disease. Such a wide range of presentations leads to diagnostic difficulties, as seen in this case. Treatment modalities include steroids, antibiotics, and surgical removal of affected parts, depending on the extent of the disease. Here, we present a case of a young male who presented with manifestations of mesenteric lymphadenitis and had an intraluminal cecal mass causing obstructive symptoms, and was subsequently diagnosed with CD.

## Introduction

Crohn's disease (CD) is one of the two major inflammatory bowel diseases (IBD), more prevalent in developed countries. The incidence of CD is 0.1-0.6 cases per 100,000 patients per year, seen equally amongst males and females [1,2]. The disease typically presents in early adulthood, with frequent abdominal pain, diarrhea, hematochezia, and weight loss. However, symptoms vary from person to person and can range from mild to severe, depending on the severity and location of the inflammation. Several genes have been studied as etiological factors implicated in CD and, thus far, strong and replicated associations have been identified with *NOD2*,* IL23R*, and *ATG16L1* genes [3]. Numerous etiologies of the disease have been proposed, including various environmental factors, autoimmunity, genetics, and gut microbiome derangement. CD can frequently aggravate and cause multiple complications, including fistulas, abscesses, obstruction, and internal bleeding. Prognostic factors for complications and a thorough series of investigations done for diagnosing CD hold significant importance in guiding therapeutic decisions. Being a chronic inflammatory condition with granulomatous inflammation, diffuse mesenteric lymphadenopathy with an intraluminal mass remains a rare initial presentation of CD.

## Case presentation

**Figure 1:**
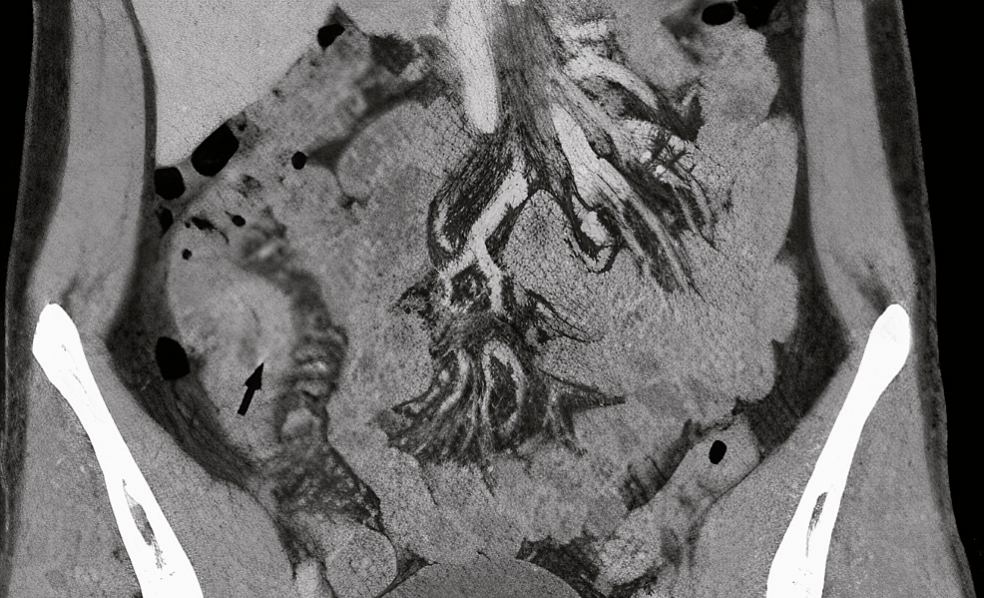
CT scan showing diffuse long segment mucosal thickening in the distal ileum, which extended over more than 12 cm. Arrow shows several enlarged lymph nodes on the right side of the bowel.

We present a case of a 25-year-old male with no significant past medical history, who presented to the emergency department (ED) with severe diffuse abdominal pain, which was colicky in nature, sudden in onset, 7/10 in intensity. He had nausea, vomiting, and loss of appetite as well. Specific investigations, including complete blood count and serum electrolytes, were carried out, and the results were normal. The patient was an avid smoker for nine years with a history of smoking one cigarette pack per day. Gastroenteritis was suspected. Thus, he was given intravenous normal saline stat, intravenous omeprazole, and intravenous baclofen in the ED and was discharged on oral medication, which included ciprofloxacin 500 mg once daily, metronidazole 400 mg thrice daily, and omeprazole 40 mg once daily for a week. One month later, he presented again to the ED with right iliac fossa pain, nausea, vomiting, loss of weight, loss of appetite, and constipation for two days. On physical examination, there was pain on deep palpation while rebound tenderness, rigidity, and guarding were absent. Investigations including complete blood count (CBC), liver function test (LFT), serum amylase, and lipase levels were ordered, and apart from mild leukocytosis and slightly elevated erythrocyte sedimentary rate (ESR), results came back normal. After reviewing labs and imaging results, a stool sample was sent for calprotectin level, which was >1000.0 ug/g, while the fecal occult blood test was negative. Computed tomography (CT) scan, as shown in Figure 1, showed diffuse long-segment mucosal thickening in the distal ileum, extending over 12cm, as well as enlarged lymph nodes in the right side of the bowel (depicted by the arrow in the figure).

**Figure 2:**
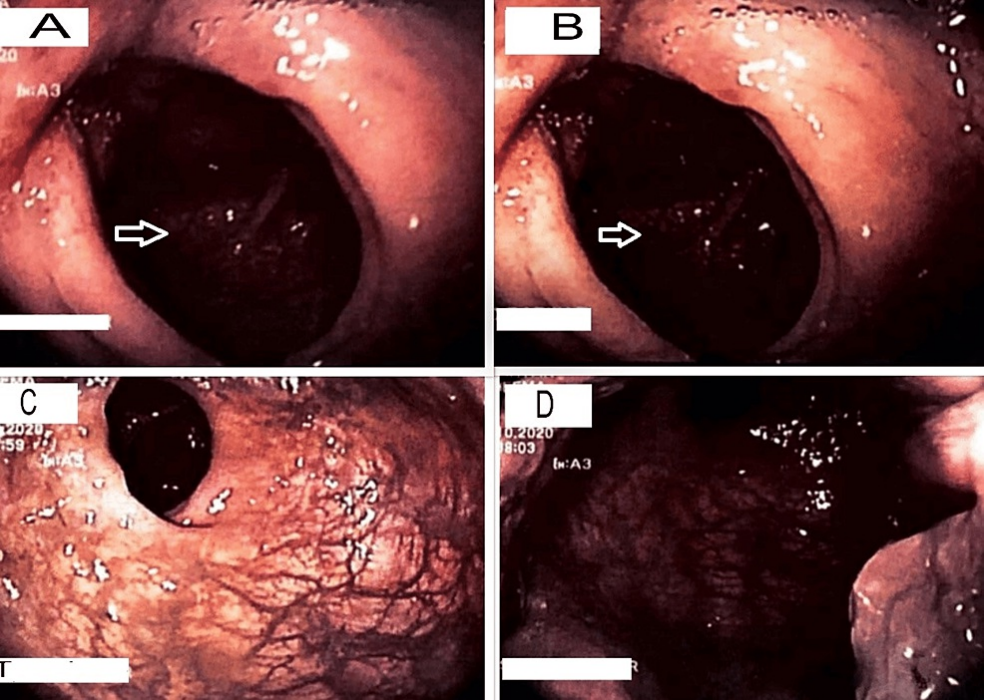
Colonoscopy showing friable mucosa (A) and (B) show the cecum and ileocecal valve, with mucosal edema and ulceration, and a completely occluded ileocecal junction. The scope could not be passed beyond this point. (C) shows the rectum and (D) shows the anorectal junction. Both are normal.

At this time, differential diagnoses included CD, intestinal lymphoma, Castleman disease, and intestinal tuberculosis. A colonoscopy done for further evaluation showed a friable, nodular ulcerated ileocecal region with complete occlusion of the ileocecal valve and surrounding mucosal edema, as shown in Figure 2 (A, B). However, the scope could not be passed beyond the ileocecal valve.

**Figure 3:**
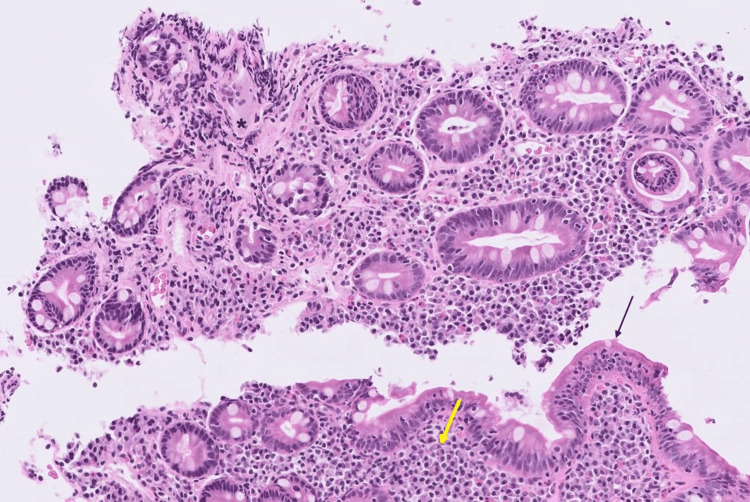
Cecum with transmural acute and chronic inflammation, focal mucosal ulceration, and rare non-necrotizing granulomas. Yellow arrow indicates an area of dense plasma cell and lymphocytic infiltrate; Black arrow indicates intraepithelial lymphocytes.

Biopsy reports did not show any specific findings. Due to the deteriorating condition of the patient, a laparoscopic modified right-sided hemicolectomy was performed. Surprisingly, a hard stony nodular mass measuring 2 cm was present at 11 cm from the proximal ileal resection margin, 7 cm from the distal colonic resection margin, and 5.3 cm from the mesenteric resection margin. The mass was predominantly located on the mesenteric border. The specimen was sent for histopathology evaluation and showed acute and chronic transmural inflammation at the cecum along with focal mucosal ulceration and rare non-caseating granulomas (Figure 3).

Twenty-one reactive mesenteric lymph nodes were noted, along with a few lymph nodes showing non-caseating granulomas. IgG and IL-6 levels were normal, and lymph node biopsy ruled out Castleman disease and lymphoma. The patient was subsequently discharged to continue outpatient follow-up for CD. 

## Discussion

CD is one of the two main types of intestinal inflammatory disorders, the other one being ulcerative colitis (UC). Transmural inflammation of the intestine is the hallmark of CD, with granulomatous features seen on biopsy [3]. This report aims to emphasize atypical symptoms and endoscopic and histopathological findings. It is important to stress that reviewing atypical presentations will be helpful in the diagnosis of such cases in the future. The colonoscopy findings of a friable, nodular ulcerated ileocecal region with complete occlusion of the ileocecal valve are not those normally seen in CD. Moreover, it is unusual for colonoscopy biopsies to be inconclusive in this disease, as ileocolonoscopy with biopsy is the gold standard for evaluating the extent and severity [4]. 

This atypical presentation of CD deviates from the most common pattern found at the time of diagnosis, where the earliest endoscopic manifestations consist of small aphthous ulcers [5], followed later by characteristic findings of skip lesions, ulcerations, and strictures [6]. The essential feature to be noted in this case is the lack of conclusive findings in all investigations conducted, including CT scan, followed by the discovery of a hard mass during laparoscopic surgery. This finding is not very common in early CD. Patients with long-standing CD can present with a large abdominal mass, and the majority of the histopathologic evaluation revealed a neoplasm [7]. An abdominal mass has also been mentioned as a possible site-specific manifestation of CD [8]. Furthermore, in cases where suspicion of both CD and UC is present, the finding of an abdominal mass in addition to nausea and colicky abdominal pain favors a diagnosis of CD [9]. The prolonged and non-self-limiting course of the disease rules out the possibility of other infectious colitis [10]. 

Considering CD as the final diagnosis, the patient was started on a six-month course of medications, which included sulfasalazine and corticosteroids. The patient presented in the outpatient department after six months for a follow-up and reported complete remission of symptoms with no acute flare-ups. In addition to the medication, cessation of smoking immensely helped in improving the prognosis. 

## Conclusions

Analyzing the journey that concluded with the diagnosis of CD in our patient, it is crucial to note that evidence is not always found on colonoscopy biopsies, and it is essential to investigate further by other findings. Though the mesenteric adenitis visible on ultrasonography, along with the symptoms of vomiting, anorexia, and weight loss, raised concerns for possible intestinal tuberculosis, with similar nonspecific manifestations in earlier stages, the histological picture of the mass ultimately overruled any such diagnosis. The diagnostic clue lies in a combined assessment of symptoms, radiology, and histological evidence. However, early aggressive treatment results in a better prognosis, especially in patients with risk factors for complications of CD. 
